# Supplementary figures and images for: Multivariate Computational Analysis of Gamma Delta T Cell Inhibitory Receptor Signatures Reveals the Divergence of Healthy and ART-Suppressed HIV+ Aging
Source: Front Immunol. 2018 Dec 5;9:2783. doi: 10.3389/fimmu.2018.02783 (PMC6290897; doi:10.3389/fimmu.2018.02783)

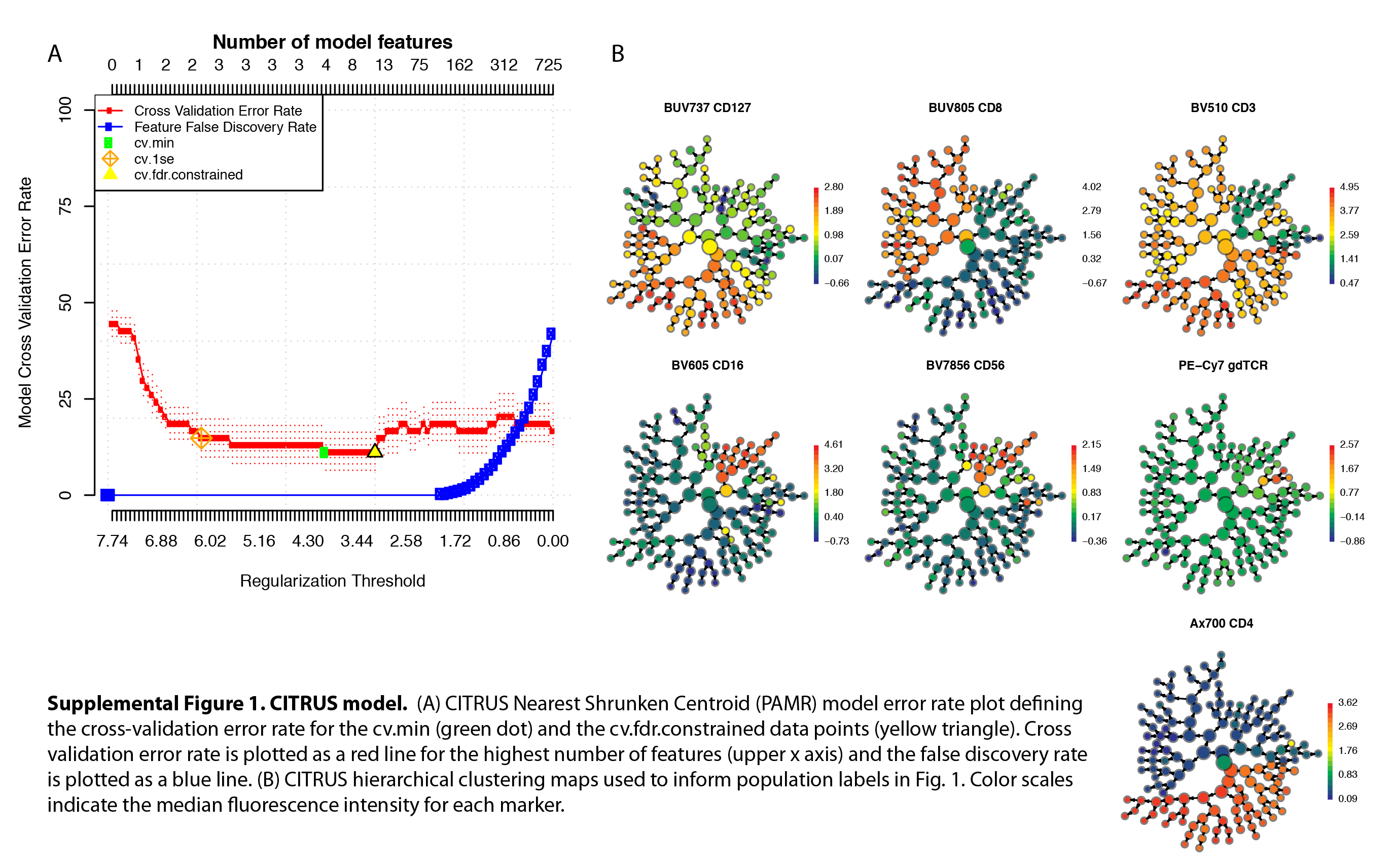

Supplement: Supplementary file 1 [file Image_1.jpg]

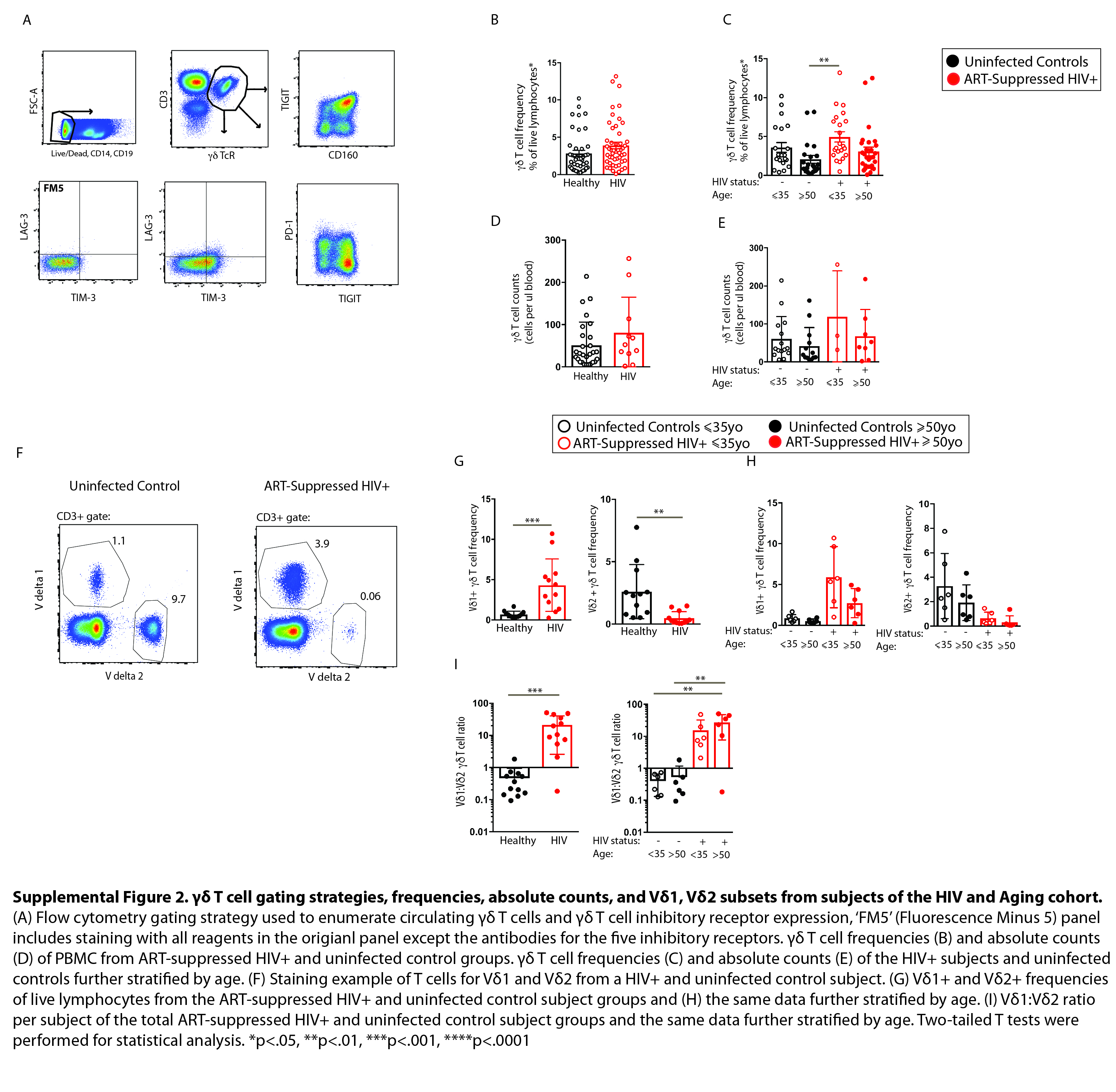

Supplement: Supplementary file 2 [file Image_2.jpg]

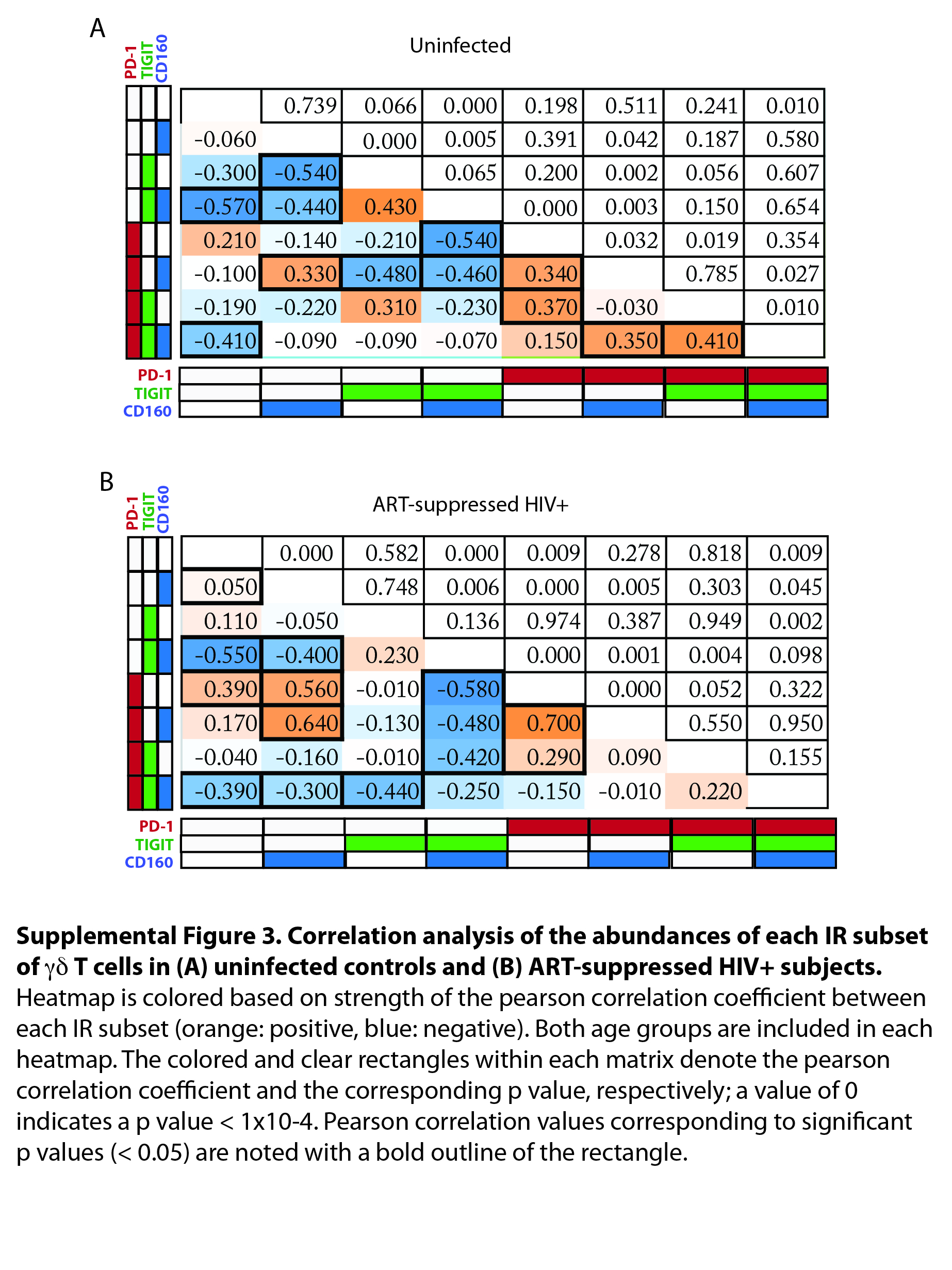

Supplement: Supplementary file 3 [file Image_3.jpeg]

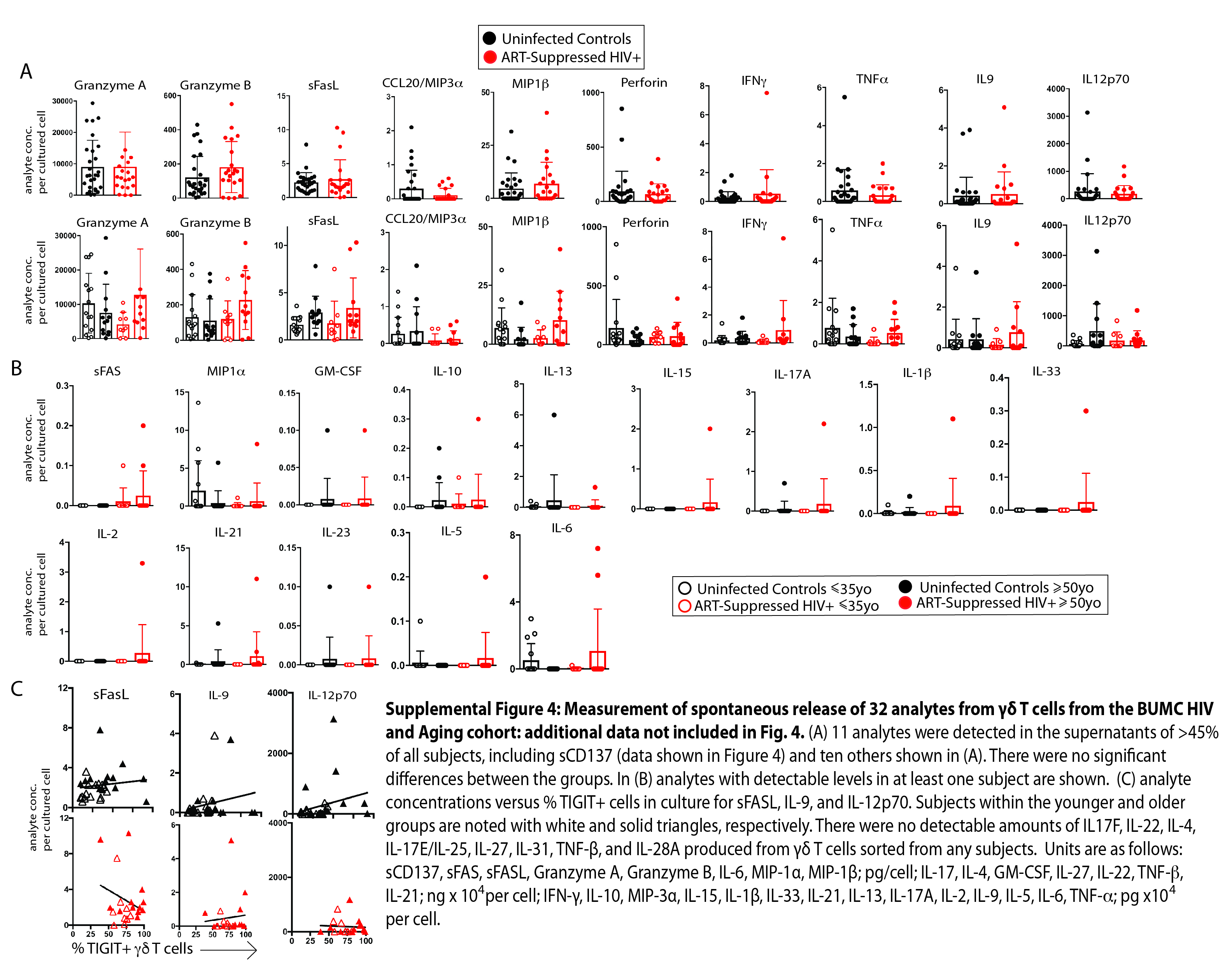

Supplement: Supplementary file 4 [file Image_4.jpg]

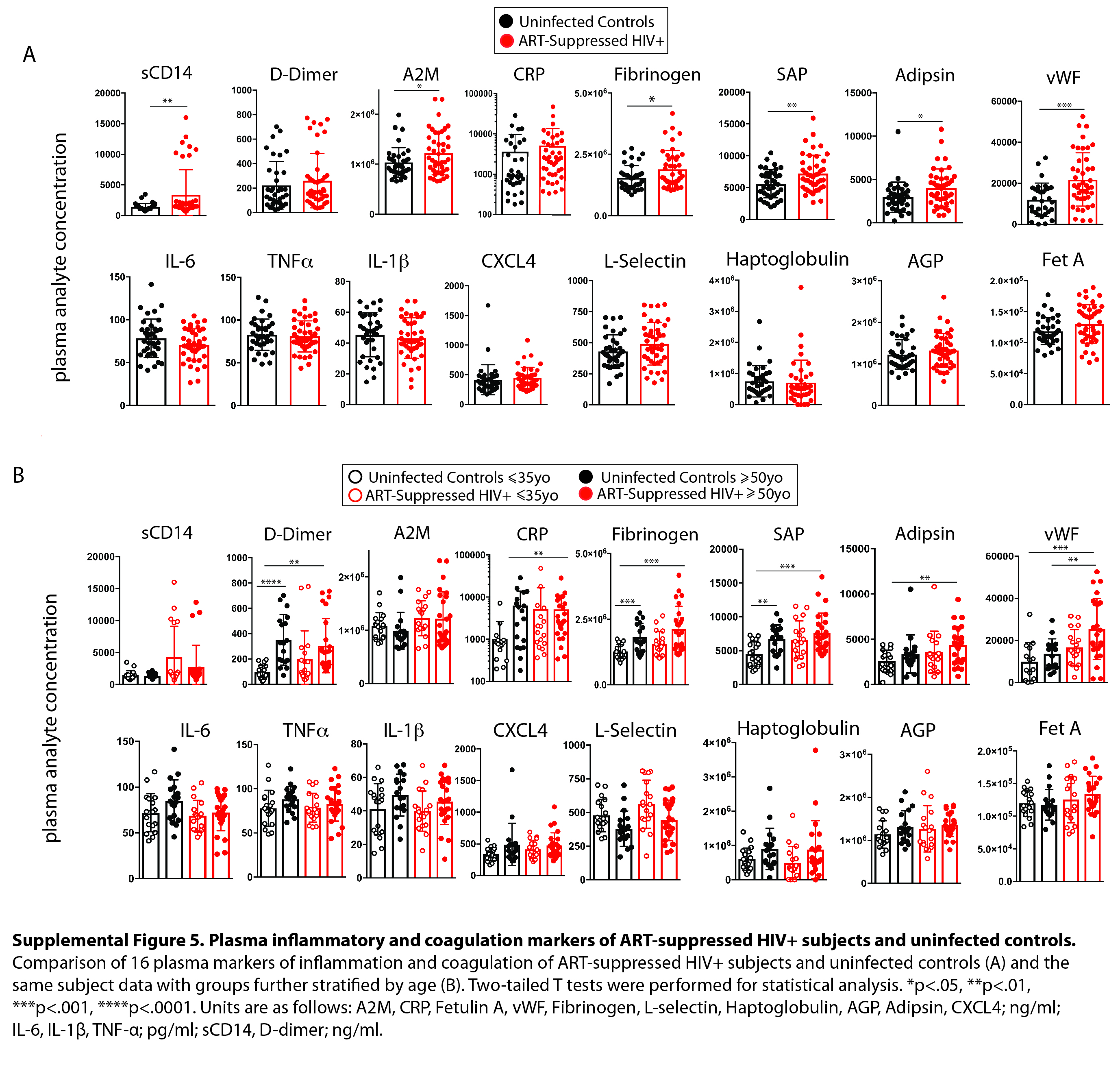

Supplement: Supplementary file 5 [file Image_5.jpg]
