# Supplementary material for: Multivariate Computational Analysis of Gamma Delta T Cell Inhibitory Receptor Signatures Reveals the Divergence of Healthy and ART-Suppressed HIV+ Aging
Source: Front Immunol. 2018 Dec 5;9:2783. doi: 10.3389/fimmu.2018.02783 (PMC6290897; doi:10.3389/fimmu.2018.02783)
Supplement: Supplementary file 6 [file Table_1.pdf]

Supplemental Table 1. Flow Cytometry Reagents

Specificity	Conjugate	Ab clone	Vendor	Panels	Catalog number
CD25	BUV 395	2A3	BD	1	564034
Streptavidin	BUV 395		BD	2	564176
CD127	BUV 737	HIL-7R-M21	BD	1	564300
CD8	BUV 805	SK1	BD	1, 2	564913
PD-1	BV 421	EH12.2H7	Biolegend	1, 2	329919
CD3	BV 510	OKT3	Biolegend	1, 2	317331
CD16	BV 605	3G8	Biolegend	1, 2	302039
Tim3	BV 650	7D3	BD	1, 2	565565
CD56	BV 786	NCAM16.2	BD	1, 2	564058
CD160	AF488	BY55	Thermo Fisher	1, 2	53-1609-41
Vα24 TCR	PE	C15	Beckman Coulter	1	COIM2283
Vδ1 TCR	PE	REA173	Miltenyi	2	130-100-536
LAG3	PE-e610	3DS223H	Thermo Fisher	1	12-2239-41
CD27	PE-e610	0323	Thermo Fisher	2	61-0279-41
TIGIT	PE-ef710	MBSA43	Thermo Fisher	1, 2	46-9500-41
γδTCR	PE-Cy7	B1	Biolegend	1, 2	331221
CD1d tetramer	APC	n/a	NIH tetramer facility	1	
Vγ9 TCR	APC	B3	Biolegend	2	331309
CD4	AF700	RPA-T4	Biolegend	1, 2	300526
CD14	APC-Cy7	HCD14	Biolegend	1, 2	325619
CD19	APC-Cy7	HIB19	Biolegend	1, 2	302217
NIR Zombie	n/a	n/a	Biolegend	1, 2	423105
Vδ2	biotin	B6	Biolegend	2	331404
Brilliant Stain Buffer	n/a	n/a	BD	1, 2	563794
Human TruStain FcX FcR Block	n/a	n/a	Biolegend	1, 2	422301
