# Supplementary material for: Multivariate Computational Analysis of Gamma Delta T Cell Inhibitory Receptor Signatures Reveals the Divergence of Healthy and ART-Suppressed HIV+ Aging
Source: Front Immunol. 2018 Dec 5;9:2783. doi: 10.3389/fimmu.2018.02783 (PMC6290897; doi:10.3389/fimmu.2018.02783)
Supplement: Supplementary file 7 [file Table_2.pdf]

Supplemental Table 2. HIV+ Subject Characteristics: statistical comparisons of common confounders between younger and older groups.

HIV+ Subject Characteristics	ART-suppressed HIV+		p-value	Total
	Younger (≤35)	Older (≥50)		
n	22	28	-	50
Age (mean, stdev)	24 (4.5)	58 (7.5)	-	45 (16.2)
Sex (% male)	100	86	0.12	92
Race			0.064	
White	11	18		29
Black	6	10		16
Asian	3	0		3
Other	2	0		2
CD4 T cells/mm^3, mean (stdev)				
Enrollment (n=41)	611 (264)	719 (311)	0.38	674 (294)
ART Start (n=44)	358 (188)	402 (283)	0.90	383 (245)
Nadir (n=49)	361 (199)	334 (220)	0.48	345 (209)
HIV-1 RNA <200 copies/mL	100%	100%	-	100%
Known Smoker			0.40	
Current	13.6%	28.6%		22%
Former	9.1%	14.3%		12%
Never	22.7%	21.4%		20%
Unknown	54.5%	35.7%		46%

		# of participants		
Test Ran	Notes	YOUNGER	OLDER	total
		22	28	50
		22	28	50
Fisher Exact test		22	28	50
Fisher Exact test				
Mann-Whitney		17	24	41
Mann-Whitney		19	25	44
Mann-Whitney		22	27	49
		22	28	50
Chi Square				
	Smoker	3	8	11
	Former	2	4	6
	Never	4	6	10
	Unknown	13	10	23
